# Procalcitonin as a biomarker in equine chronic pneumopathies

**DOI:** 10.1186/s12917-016-0912-4

**Published:** 2016-12-09

**Authors:** Ann Kristin Barton, Anna Pelli, Martin Rieger, Heidrun Gehlen

**Affiliations:** 1 Equine Clinic, Freie Universitaet Berlin, Oertzenweg 19b, 10163 Berlin, Germany; 2 Research unit microbe-plant interactions, Helmholtz Zentrum Muenchen, Ingolstädter Landstraße 1, 85764 Neuherberg, Germany

**Keywords:** Horse, Recurrent airway obstruction, Inflammatory airway disease, Chronic interstitial pneumopathy, Procalcitonin, Biomarker

## Abstract

**Background:**

Procalcitonin (PCT), a precursor protein of the hormone calcitonin, is a sensitive inflammatory marker in human medicine, which is primarily used for diagnosis of bacterial sepsis, but is also useful in diagnosis of exacerbation of asthma and COPD. In this study, PCT was evaluated as a potential biomarker for different chronic pneumopathies in the horse using an equine specific ELISA in comparison to established clinical markers and different interleukins.

Sixty-four horses were classified as free of respiratory disease, recurrent airway obstruction (RAO), inflammatory airway disease (IAD) or chronic interstitial pneumopathy (CIP) using a scoring system. PCT concentrations were measured in plasma (*n* = 17) and in the cell-free supernatant of bronchoalveolar lavage (*n* = 64). PCT concentrations were correlated to interleukins IL-1ß and IL-6 in BALF, clinical findings and BALF cytology.

**Results:**

The median PCT concentrations in plasma were increased in respiratory disease (174.46 ng/ml, *n* = 7) compared to controls (13.94 ng/ml, *n* = 10, *P* = 0.05) and correlated to PCT in BALF supernatant (rs = 0.48). Compared to controls (5.49 ng/ml, *n* = 15), median PCT concentrations in BALF supernatant correlated to the overall clinical score (rs = 0.32, *P* = 0.007) and were significantly increased in RAO (13.40 ng/ml, *n* = 21) and IAD (16.89 ng/ml, *n* = 16), while no differences were found for CIP (12.02 ng/ml, *n* = 12). No significant increases were found for IL-1 and IL-6 between controls and respiratory disease in general as well as different disease groups.

**Conclusions:**

Although some correlations were found between PCT in plasma, BALF supernatant and clinical scores, PCT in BALF does not seem to be a superior marker compared to established clinical markers. PCT in plasma seems to be more promising and a greater number of samples should be evaluated in further studies.

## Background

Since the 1970s, hypocalcemia and its correlation to sepsis have been in the focus of research [1]. Procalcitonin (PCT) is a precursor protein of the hormone calcitonin, which regulates the calcium homeostasis by inhibition of osteoclastic activity. In health, preprocalcitonin (prePCT) is exclusively produced in the thyroid c-cells. Low concentrations of <0.1 ng/ml are found in human serum [2].

During sepsis, PCT is found in high concentrations in blood and almost all tissues [3], but PCT is not only a precursor of calcitonin leading to hypocalcemia. It seems to have another pathophysiologic role as an inflammatory mediator and is found in almost all inflammatory processes independent from hypocalcemia. Its synthesis can be triggered by TNF-α and several interleukins [4, 5]. Despite lower PCT concentrations in plasma compared to endotoxemia and sepsis, differentiation between different forms of pneumonia is possible [6–8] and chronic respiratory diseases like asthma or COPD (chronic obstructive pulmonary disease) are also characterized by increases in PCT concentrations. As acute exacerbation of asthma is often caused by bacterial infections of the lower airways, PCT can be used for the decision pro or contra the initiation and duration of antibiotic therapy [9] and can help in the interpretation of indifferent thoracic radiographs [10]. Similar results have been found for COPD, where PCT measurement can support the decision for individual therapy involving antibiotics or glucocorticoids and is considered to be helpful in long-term management [11, 12].

Equine RAO (recurrent airway obstruction) is known for parallels to human asthma (respiratory hypersensitivity, good response to bronchodilators and glucocorticoids) as well as COPD (airway neutrophilia, epithelial metaplasia and hypersecretion into the lower airways), resembling what is called “wheezy bronchitis” in men [13]. During disease exacerbation, increased inflammatory markers may be found in plasma as described for haptoglobin and serumamyloid A [14], but local neutrophila in bronchoalveolar lavage fluid (BALF) is considered the most reliable feature [15]. Inflammatory airway disease (IAD), a possible precursor of RAO [16], shows a lower grade of inflammation including airway neutrophilia and/or increases in mast cell and eosinophil counts [17], while interstitial pneumopathies are characterized by increases in macrophage percentages in BALF cytology [18, 19]. As systemic markers were only increased in RAO exacerbation, measurement of biomarkers may be more rewarding out of BALF in chronic respiratory disease. High disease prevalence and economic impact have been described for equine chronic respiratory disease, therefore new biomarkers may help to differentiate these pneumopathies and to identify cases of subclinical disease. A species specific ELISA for equine PCT (ePCT) was established [20], which allows quantitative measurements instead of gene expression with a high specificity and sensitivity. High ePCT concentrations were found in plasma of endotoxemic horses suffering from colic [21, 22].

In this study, we aimed to compare ePCT concentrations in BALF with clinicals findings, BALF cytology and other biomarkers, namely interleukins 1ß and 6, which have been shown to stimulate hyperprocalcitonemia in other species [4, 23]. Additionally, ePCT concentrations were measured in plasma. We hypothesized that ePCT correlates with clinical scores, BALF cytology and interleukins in RAO, IAD and CIP and may be a superior marker in cases of low-grade inflammation.

## Methods

### Preparticipation examination

A total of 71 horses were examined, of which 15 had no clinical signs or history of respiratory disease and 56 were presented to the clinic with a history of chronic lower airway disease.

The pre-participation examination included anamnesis documentation, clinical examination, exercise test, blood gas analysis, bronchoscopy, BALF cytology and thoracic radiography. Using a modified clinical score system [24, 25], endoscopy score results, parameters of gas exchange, BALF cytology and thoracic radiography results, horses were classified as free of respiratory disease (controls, group I), Recurrent airway obstruction in exacerbation (RAO, group II), Inflammatory airway disease (IAD, group III), chronic interstitial pneumopathy (CIP, group IV) or were excluded from the study, if they could not be assigned to these groups (*n* = 7). In detail, groups were defined as follows:

- Group I: No history of respiratory disease, clinical score < 2, no tracheal secretions, low cellular density and neutrophils ≤ 8% in BALF, AaDO_2_ ≤ 8 mmHg, exclusion of acute signs of infection (leukocytosis, fever, depression).
- Group II: History of recurrent cough or dyspnea, clinical score > 6, high amount or viscosity of tracheal secretions, high cellular density and neutrophils ≥ 25% in BALF, AaDO_2_ > 8 mmHg, exclusion of acute signs of infection (leukocytosis, fever, depression) according to *Robinson* [15].
- Group III: History of cough or exercise insufficiency, clinical score 2–6, low to moderate amount or viscosity of tracheal secretions, increased cellular density and neutrophils ≥ 8% or mast cells ≥ 2% or eosinophils ≥ 0.1% in BALF, exclusion of acute signs of infection (leukocytosis, fever, depression) according to *Couëtil* et al. [17].
- Group IV: History of exercise insufficiency, clinical score 2–6, low to moderate amount or viscosity of tracheal secretions, increased cellular density and ratio of macrophages:neutrophils ≥ 2.5:1 in BALF, increased interstitial opacity of thoracic radiographs, exclusion of acute signs of infection (leukocytosis, fever, depression) according to *Dieckmann* et al. [18].

### BALF collection and processing

During endoscopy, 20 ml of 2% lidocaine (Bela-Pharm GmbH, Vechta, Germany) were infused around the tracheal bifurcation. The catheter (Silicone Bronchoalveolar Lavage Catheter 300 cm, Smiths Medical ASD, Inc, USA) was wedged into the bronchus by mean of an air balloon. Five hundred milliliters of pre-warmed phosphate buffered saline (Phosphate buffered saline, Lonza, Verviers, Belgium) were infused as recommended by the International Workshop on Equine Chronic Airway disease [15] and immediately aspirated.

BALF was divided into 2 portions for cytological and biochemical examination. After centrifugation at 1500 rpm for 10 min at 4 °C the cell-free supernatant was stored at −80 °C until assayed. Cytology was performed using Wright-Giemsa staining and counting 500 cells at 500× magnification.

### ELISA for quantification of ePCT in plasma and BALF

As the ELISA for equine PCT was only validated for plasma, it was adapted to BALF according to the guidelines of the American Food and Drug Administration for Validation of bioanalytic methods (2013) by partial validation which was done here by reanalyzing intra-assay (IaA) and inter-assay (IeA) coefficients of variation (CV) and recovery in BALF matrix (Table 1).

**Table 1:** Concentrations spiked (Conc_spik_) and found (Conc_found_), intra-assay (IaA) and inter-assay (IeA) coefficients of variation (CV) and recovery-values for equine PCT ELISA in BALF

Sample	Conc_spik_[ng/ml]	Conc_found_[ng/ml]	IaA-CV (%)	IeA-CV (%)	Recovery (%)
1	10	15.6 ± 4.7	30.4	10.9	156
2	**25**	24.9 ± 0.7	**2.7**	**8.4**	**99.2**
3	**50**	43.2 ± 2	**4.7**	**3.1**	**86.2**
4	**100**	102.6 ± 13.9	**13.6**	**11.4**	**102.5**
5	**250**	377 ± 22.3	**5.9**	**7**	**150.8**
6	500	658.5 ± 248	37.7	16.1	131.6
7	1.000	*	*	*	*
8	5.000	*	*	*	*
9	10.000	*	*	*	*

The working range is marked in bold

### Measurements of PCT in plasma

In 17 horses (10 controls, 7 horses with respiratory disease), PCT was measured in plasma using a specific ELISA for equine PCT [20]. As this was not part of the original study, we did not have plasma samples available from all horses.

### Measurements of PCT in BALF

In 64 horses, PCT was measured in undiluted BALF supernatant samples using a specific ELISA for equine PCT [20] after adaption to this substrate.

### Measurements of interleukins

In 64 horses, interleukins was measured in undiluted BALF samples using commercially available specific ELISAs for equine IL-1ß and 6 (ELISA Kit for Interleukin 1 Beta (IL1b) and ELISA Kit for Interleukin 6 (IL6), Uscn Life Science Inc, USA) according to the manufacturer’s manual.

#### Statistical analysis

Data were statistically analyzed using SPSS (SPSS Statistics, Version 17.0 released 2008, SPSS Inc., USA) and expressed as median (min-max). The data were tested for normal distribution using the Kolmogoronov-Smirnov and Shapiro Wilks Test. The level of significance was set at *P* ˂0.05.

Kruskal Wallis H test was used to compare between controls and different disease groups followed by Post-Hoc testing using Mann-Whitney *U* Test for 2-group comparison to determine intergroup differences.

Spearman rank correlation coefficients were calculated between clinical parameters, cytologic data, PCT and interleukin concentrations. Correlations were classified as irrelevant (rs = 0–0.25), weak (rs = 0.25–0.5), moderate (rs = 0.5–0.75) and strong (rs > 0.75).

## Results

### Clinical scoring

According to the results of clinical scoring and BALF cytology, the 71 horses (42 geldings, 29 mares, age 12.5 ± 5.31 years, BDW 470.8 ± 91.3 kg) presented for participation in this study were classified as follows: 15 horses were classified as free of respiratory disease (group I, controls), 21 as RAO (group II), 16 as IAD (group III), 12 as chronic interstitial pneumopathy (group IV), 4 horses suffered from acute respiratory infections and 3 could not be clearly assigned to groups I-IV. Therefore, the later 7 were excluded, leaving 64 horses for statistical analysis.

### Hematology and ionized calcium

In all 64 horses, controls and horses affected by respiratory disease, hematology was unremarkable. Leucocytes (8.1 ± 2.2 * 10^3^/μl), hematocrit (35 ± 5.1%), total protein (6.7 ± 0.5 g/dl) and ionized calcium (1.64 ± 0.10 mmol/l) were within normal limits. There was no difference between in ionized calcium between controls (1.58 ± 0.11 mmol/l) and horses affected by respiratory disease in groups II-IV (1.65 ± 0.09 mmol/l).

### Measurements of PCT in plasma

In 17 horses (10 controls, 7 affected by respiratory disease), PCT was measured in plasma using a specific ELISA for equine PCT [20]. Overall PCT concentrations in respiratory disease (174.46 ng/ml, *n* = 7) were higher than in group I (controls, 13.94 ng/ml, *n* = 10). The median concentrations in group II (RAO) was even 482.23 ng/ml (*n* = 4).

### Adaption of PCT ELISA to BALF

According to the guidelines of the *American Food and Drug Administration for Validation of Bioanalytic Methods* (2013) the variation coefficients in medical ELISAs need to be below 15%, therefore the working range for equine BALF was defined as 25–250 ng/ml, as shown in Table 1.

### Measurements of PCT in BALF

**Fig. 1:**
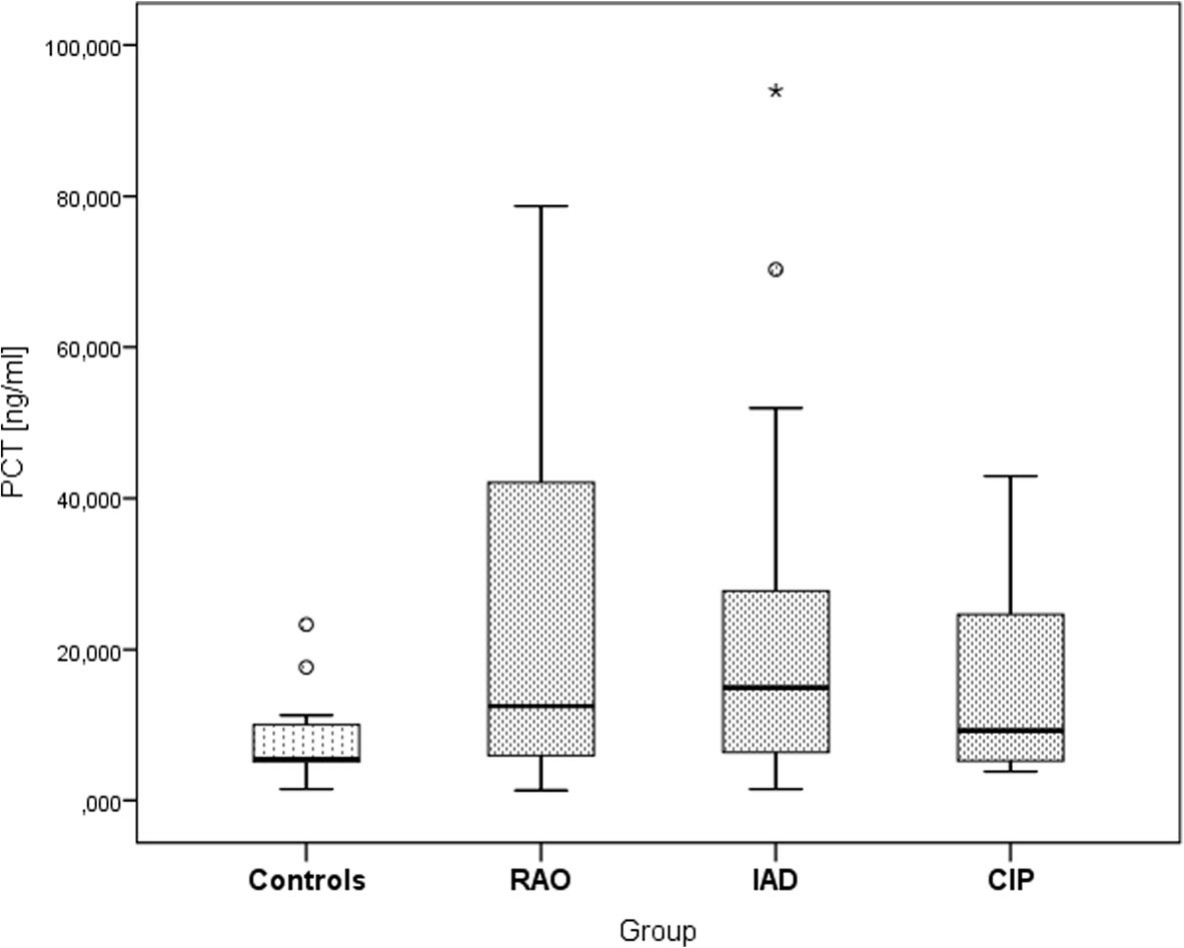
PCT concentrations (0–250 ng/ml) in BALF over different disease groups (*n* = 59). Five samples had PCT values > 250 ng/ml and were excluded from this graph, but not from statistical analysis. ° shows outliers 1.5–3 times out of the interquartile range, * shows extreme outliers > 3 times out of the interquartile range

In 64 horses, PCT was measured in undiluted BALF supernatant using a specific ELISA for equine PCT [20] after adaption to this substrate. The median concentration in group I (controls, 5.49 ng/ml) as well as in groups II-IV was below the defined working range of the ELISA (25–250 ng/ml). PCT was significantly increased in groups II (RAO, median13.40 ng/ml) and III (IAD, median 16.89 ng/ml), while no differences were found for group IV (CIP, median 12.02 ng/ml), Table 2 and Fig. 1. Significant inter-group differences were found between groups I (controls) and II (RAO, *P* = 0.033) as well as groups I and III (IAD, *P* = 0.006).

**Table 2:** Median values of PCT and interleukin concentrations in BALF supernatant over different disease groups, significance (*P* < 0.05) is indicated by*

Diagnosis	PCT [ng/ml]	IL-1β [pg/ml]	IL-6[pg/ml]
Controls (*n* = 15)	5.49 (1.49–23.32)	5.7 (5.7–104)	2.5 (2.5–25)
RAO (*n* = 21)	13.40* (1.32–681.66)	25 (5.7–122)	11 (2.5–53)
IAD (*n* = 16)	16.89* (1.49–493.72)	22 (5.7–84)	3.5 (2.5–41)
CIP (*n* = 12)	12.02 (3.82–1761.86)	33.5 (5.7–121)	10.5 (2.5–63)

### Correlation of PCT to clinical parameters

A weak correlation was found between PCT concentrations in plasma and BALF supernatant (rs = 0.48, *P* = 0.060). The highest correlation was found between PCT in plasma and the respiratory rate (rs = 0.53, *P* = 0.025). Negative correlations were found between PCT in plasma and the arterial PaO_2_ (rs = −0.60, *P* = 0.009). A weak correlation was found between PCT concentration in BALF and airway obstruction (rs = 0.42, *P* = 0.010) as well as overall clinical score (rs = 0.32, *P* = 0.007). Neither PCT in plasma nor in BALF supernatant correlated to the percentage of neutrophils in BALF cytology, but PCT in plasma with the eosinophils (rs = −0.49, *P* = 0.039).

### Measurements of interleukins in BALF

In 64 horses, interleukin concentrations were measured in undiluted BALF samples using a specific ELISA for equine IL-1ß and IL-6. No significant increases were found for IL-1ß or IL-6 between group I (controls) and groups II-IV in general (*P* = 0.122) or between controls and single disease groups (*P* = 0.365). Results are shown in detail in Table 2.

## Discussion

In the late 1990s, it has been shown that procalcitonin is a sensitive marker for systemic inflammatory processes caused by bacterial infection, but not of other pathogenesis [26–28]. While exacerbations of asthma and COPD in men are often triggered by viral and bacterial infection of the lower airways [29, 30], this is not a common feature in RAO [31]. In IAD, however, microbiologic examinations of tracheal aspirates are often positive despite no evidence of systemic infection [32]. Interstitial pneumonia may also be the consequence of bacterial infection, but in the phase of chronic interstitial disease it is usually impossible to find evidence of bacterial disease [19].

Equine PCT was characterized in the early 2000s [33]. Hypocalcemia in case of acute systemic disease was also found in horse [34]. Low plasma levels of ionized calcium were found in severe colic, gastrointestinal inflammation and induced endotoxemia [35, 36]. Studies in horses suffering from acute or chronic respiratory disease have not been published so far. In our study, concentrations of ionized calcium were within normal limits, both in healthy controls and in horses affected by chronic pneumopathies. Of course, chronic respiratory disease is not commonly associated with endotoxemia, but ePCT may have a pro-inflammatory function independent from its role in calcium metabolism, possibly playing a role in chronic respiratory disease. It was shown in other species that anti-PCT antibodies increase the chance of survival after induced endotoxemia [37–39].

For the quantification of ePCT in plasma and BALF a validated sandwich ELISA using human anti-PCT Antibodies and equine recombinant PCT (erPCT) as standard was used [20]. Rieger et al. [20] showed that the assay has a working range of 25–1000 ng/mL. The authors stated parallelism up to a concentration of 10.000 ng/mL. Higher concentrations of ePCT led to irregularities in quantification regarding different dilutions. However, the authors showed acceptable values for IaCv and Recovery resulting in a working range of 25–1000 ng/mL. Changing the matrix for validated immunoassays can be done by partial validation which was done here by reanalyzing IaCV/IeCV and recovery in BALF matrix. Although the working range was defined to be 25–250 ng/mL, lower concentrations can be reported with this assay, but results must be regarded with caution, as variability at 10 ng/mL (which was approximately the BALF PCT concentration within this study) is higher (30%). For future studies in BALF, the method has to be improved e.g., by immunoaffinity enrichment of ePCT before analyzing the samples in the assay or the development of a more sensitive method, for example electrochemiluminescence (ECl).

In human medicine, PCT concentrations are routinely measured systemically from blood samples. A good correlation has been found between PCT in serum and BALF [40, 41]. Studies focusing on PCT in BALF samples only, however, have led to disappointing results. In some cases, very low concentrations were found in the alveolar space making it impossible to use PCT in the differentiation of pulmonary disease [40–44]. Low sensitivity of methods of detection and the dilution effect of BALF may have been the reasons for this [40, 43]. In equine medicine, bronchoalveolar lavage is an easy to perform and safe procedure to characterize airway inflammation and is recommended for the diagnosis of common respiratory disease like RAO or IAD [15, 17]. Much higher ePCT concentrations were found in serum of horses compared to men [20]. Therefore, it seemed logical that alveolar concentrations might also be higher in this species. Several biomarkers have been evaluated in equine BALF, as it has been shown that chronic respiratory disease does not always have a systemic component and inflammatory processes may be localized to the lung in case of minor disease like RAO in remission [14]. As PCT in plasma is only increased in case of systemic inflammation [45], we suspected that increases in ePCT would be most obvious in the alveolar space, but the results of our study show that it might be better to measure ePCT concentrations in plasma. This might be an advantage, as blood samples are much easier to obtain than BALF, but should be confirmed in a higher number of samples, as correlations based on different numbers of samples have to be interpreted with caution. ePCT in BALF allowed differentiating between controls and chronic respiratory disease, while no differentiation was possible between different disease groups. The highest concentrations were found in RAO in exacerbation and IAD, but inter-group-differences were insignificant. Again, a higher number of samples may allow to establish reasonable cut-off values for different types of disease.

A week positive correlation was found between PCT and clinical score. In human asthma, a correlation between PCT and the severity of disease has also been described [9]. Pro-inflammatory cytokines including IL-1ß and IL-6 are involved in stimulation of PCT synthesis [4, 46]. As these mediators also play a role in acute and chronic pulmonary disease, we evaluated their concentration and correlation to PCT in equine BALF. Nevertheless, no correlation was found between PCT and these interleukins in our study. Therefore, our results do not support their role in triggering PCT synthesis in the alveolar space.

For IL-1ß, no significant differences were found between healthy controls and horses affected by chronic respiratory disease in general and single disease groups, while in former studies increased transcription and protein concentrations have been found in severe RAO [47–50]. Similar results have been found for IAD [51, 52] and human asthma and COPD [53, 54]. In our study, the highest concentrations were found in CIP, where IL-1ß is known to stimulate the fibronectin and collagen synthesis. Therefore, it is a direct mediator of pulmonary fibrosis formation [54]. IL-1ß is produced by activated macrophages, which are the dominant cell type in BALF cytology in CIP.

For IL-6, no significant differences were found between healthy controls and horses affected by chronic respiratory disease either. The highest median concentration was found in group II (RAO in exacerbation). Former studies have led to contrasting results. While some authors found no significant increases in IL-6 were found in RAO exacerbation [50, 55, 56], others describe increases about three days after natural challenge [48, 52].

A weak point of this study was group definition, as samples were obtained from clinic patients. Therefore disease status was not as uniform as in experimental studies, where samples were obtained after defined disease induction. On the other hand, biomarkers are used in a clinical setting, therefore it seems logical to evaluate their usefulness in the same situation. Although IAD and RAO in remission were planned as two distinct groups, it was not possible to differentiate clearly between these two. Anamnestic information of recurrent respiratory distress was often unreliable and the majority of owners did not agree to a natural challenge test. Descriptions of equine CIP are rare in the literature and an international consensus statement is missing, so definition of this group was based on a quite old clinical case series including only 12 horses [18]. Thoracic radiography showing interstitial patterns is not very specific for CIP and may also be found in RAO and IAD [57, 58]. Again, the majority of owners did not agree to lung biopsies. We tried to face these problems by calculating correlations between PCT and interleukins with clinical and cytologic parameters over all 64 horses independent of diagnosis.

## Conclusions

In conclusion, correlations between PCT concentrations in plasma and BALF supernatant were found and also with clinical data, but PCT in BALF does not seem to be a superior marker compared to established clinical markers, in particular in cases of low-grade inflammation. Following the definition by Couëtil [17], a lower grade of inflammation can be assumed in IAD compared to RAO, but PCT was also not able to differentiate between these two. There were significant differences between controls and respiratory disease in general, but differentiation between different forms of chronic pneumopathies was not possible. PCT in plasma seems to be a more promising marker, but its usefulness should be evaluated in a higher number of cases.

## Abbreviations

AaDo_2_: Arterio-alveolar pressure difference
BALF: Bronchoalveolar lavage fluid
BDW: Body weight
CIP: Chronic interstitial pneumopathy
COPD: Chronic obstructive pulmonary disease
ELISA: Enzyme-linked immunosorbent assay
IAD: Inflammatory airway disease
IL: Interleukin
LaGeSo: State Office of Health and Social Affairs Berlin
PCT: Procalcitonin
RAO: Recurrent airway obstruction
Rpm: Rounds per minute
SD: Standard deviation
TNF-α: Tumer-necrosis-factor α
